# Physiological and Biochemical Analyses Shed Light on the Response of *Sargassum vulgare* to Ocean Acidification at Different Time Scales

**DOI:** 10.3389/fpls.2017.00570

**Published:** 2017-04-19

**Authors:** Amit Kumar, Hamada AbdElgawad, Immacolata Castellano, Maurizio Lorenti, Massimo Delledonne, Gerrit T. S. Beemster, Han Asard, Maria Cristina Buia, Anna Palumbo

**Affiliations:** ^1^Center of Villa Dohrn–Benthic Ecology, Department of Integrative Marine Ecology, Stazione Zoologica Anton DohrnNaples, Italy; ^2^Integrated Molecular Plant Physiology Research Group, Department of Biology, University of AntwerpAntwerp, Belgium; ^3^Department of Biology and Evolution of Marine Organisms, Stazione Zoologica Anton DohrnNaples, Italy; ^4^Department of Biotechnology, University of VeronaVerona, Italy

**Keywords:** macroalgae, ocean acidification, *Sargassum vulgare*, CO_2_ vents, transplants

## Abstract

Studies regarding macroalgal responses to ocean acidification (OA) are mostly limited to short-term experiments in controlled conditions, which hamper the possibility to scale up the observations to long-term effects in the natural environment. To gain a broader perspective, we utilized volcanic CO_2_ vents as a “natural laboratory” to study OA effects on *Sargassum vulgare* at different time scales. We measured photosynthetic rates, oxidative stress levels, antioxidant contents, antioxidant enzyme activities, and activities of oxidative metabolic enzymes in *S. vulgare* growing at a natural acidified site (pH 6.7) compared to samples from a site with current pH (pH 8.2), used as a control one. These variables were also tested in plants transplanted from the control to the acidified site and *vice-versa*. After short-term exposure, photosynthetic rates and energy metabolism were increased in *S. vulgare* together with oxidative damage. However, in natural populations under long-term conditions photosynthetic rates were similar, the activity of oxidative metabolic enzymes was maintained, and no sign of oxidative damages was observed. The differences in the response of the macroalga indicate that the natural population at the acidified site is adapted to live at the lowered pH. The results suggest that this macroalga can adopt biochemical and physiological strategies to grow in future acidified oceans.

## Introduction

Marine macroalgae are a large and diverse group of photoautotrophs that contribute significantly to global primary production and to blue carbon sequestration (Krause-Jensen and Duarte, 2016). In addition, canopy-forming macroalgae play important roles in structuring and sustaining biodiversity and ecosystem functioning because they modify the physical environment, provide shelter, food, breeding grounds, and nurseries for a large number of associated species, such as invertebrates and fishes (Anderson, 1994; Edgar et al., 2004; Arkema et al., 2009; Cárdenas et al., 2016). Living in coastal marine environments, macroalgae often face harsh conditions, due to temperature and salinity variations, light exposure, UV radiation, desiccation and wave action. In the last decade, an additional stress arising from rapid global climate change, causing ocean acidification (OA), has been shown to affect algal physiology, life cycles, community structures and dynamics (Harley et al., 2012; Bradassi et al., 2013; Kroeker et al., 2013a; Porzio et al., 2013; Ji et al., 2016).

Ocean acidification is a shift in seawater pH due to the rising CO_2_ concentrations in the atmosphere and in the oceans. Theoretically, elevated CO_2_ stimulates photosynthesis, and therefore OA should benefit marine autotrophs (Koch et al., 2013). However, marine plants show variable responses, related to contrasting uptake mechanisms for dissolved inorganic carbon (DIC) they possess (Mackey et al., 2015). In seawater, three major forms of DIC exist: aqueous CO_2_ at low percentage (1%), carbonate (CO_3_^2-^), and the most abundant bicarbonate ${\mathrm{HCO}}_{3}^{–}$ (92%) (Beer et al., 2014). All macroalgae can utilize aqueous CO_2_ via diffusion; however, this mechanism is around 10,000 times slower in water than in air (Ji et al., 2016). Therefore, this rate limits the supply of CO_2_ for photosynthesis (Cornwall et al., 2012). Hence, macroalgae have developed active and efficient carbon concentrating mechanisms (CCMs) that utilize ${\mathrm{HCO}}_{3}^{–}$ at the expense of high energy investments (Koch et al., 2013) to allow photosynthesis under a wide range of environmental conditions (Raven et al., 2014). When the concentration of CO_2_ increases, species with CCM may benefit from increased acidity by shifting their carbon source from ${\mathrm{HCO}}_{3}^{–}$ to CO_2_, and reduce the energy costs (Wu et al., 2008). However, macroalgae with active CCMs show contrasting responses: no enhanced production, growth or change in RuBisCO activity (e.g., the green algae *Ulva* sp., *Enteromorpha linza*; the red algae *Gracilaria conferta, Porphyra* sp., *Hypnea musciformis, Hypnea cornuta, Pterocladia capillaceae, Gelidium crinale*, and *Soliieria* sp.; the brown algae *Cystoseira* sp., *Padina pavona*, and *Sptaglossum* sp.; Israel and Hophy, 2002), or increased growth and photosynthesis (the red macroalgae *Gracilaria* sp. and *G. chilensis*; Gao et al., 1993; Wu et al., 2008). Macroalgae lacking CCMs are more likely to be carbon-limited and thus to benefit from additional CO_2_ (e.g., red macroalgae *Lomentaria articulata*; Kübler et al., 1999). Therefore, non-calcifying algae, lacking CCMs, are expected to respond positively to increasing global CO_2_ concentrations (Kroeker et al., 2010, 2013b; Harley et al., 2012). Consistently, among autotrophs, calcifying macroalgae were the most vulnerable group to the reduction of calcium carbonate saturation under future ocean conditions (Martin et al., 2008; Martin and Gattuso, 2009; Porzio et al., 2011; Harley et al., 2012; Kroeker et al., 2013b; Hofmann and Bischof, 2014).

A higher CO_2_ concentration could also affect other physiological processes, including reproduction, ion homeostasis, energy metabolism, and nutrient uptake (Roleda et al., 2012; Hofmann et al., 2013; Gutow et al., 2014; Fernández et al., 2015; Xu et al., 2015; Nunes et al., 2016; Leal et al., 2017). Moreover, these effects could be further affected by synergistic interactions with changes in other environmental factors (i.e., light and temperature; Xu et al., 2010; Zou et al., 2011; Celis-Plá et al., 2015).

Environmental changes often affect the production of reactive oxygen (ROS) and nitrogen (RNS) species in macroalgae (Dring, 2005; Kumar et al., 2015). The accumulation of ROS due to the imbalance between the production of oxidants and antioxidants leads to oxidative stress (Møller et al., 2007). Under these conditions, ROS likely oxidize proteins, lipids, and nucleic acids, thus causing cellular dysfunctions (Carvalho et al., 2004). ROS also act as signaling molecules altering gene expression and modulating the activity of specific defense proteins (Tripathy and Oelmüller, 2012). Elevated CO_2_ can induce oxidative stress in marine organisms by increasing ROS production either directly by increased formation of free radicals, due to the interaction of CO_2_ with other ROS, and/or indirectly by enhancing Fenton reaction at lower pH (Tomanek et al., 2011; Hu et al., 2015). The possible induction of oxidative stress in autotrophs by OA has been poorly investigated and mostly limited to phytoplankton, where CO_2_/lowered pH has been shown to induce oxidative stress (Brutemark et al., 2015; Yangüez et al., 2015). Elevated CO_2_ has been also shown to alleviate high PAR and UV stress in the unicellular chlorophyte *Dunaliella tertiolecta* (Garcia-Gomez et al., 2014). However, it has been demonstrated that the capacity of macroalgae to survive stress conditions is correlated with their ability to detoxify the ROS by antioxidant defense systems (Davison and Pearson, 1996). These systems include non-enzymatic (e.g., tocopherol, ascorbate, polyphenols, carotenoids) and enzymatic components [e.g., superoxide dismutase (SOD), catalase (CAT), ascorbate peroxidase (APX), glutathione peroxidase (GPX), glutathione *S*-transferase (GST); Bischof and Rautenberger, 2012].

Most of our understanding of the effect of OA on macroalgae is obtained in confined short-term studies. In contrast to natural environments, such studies mainly involve a single species and conditions of constant and stable carbonate chemistry parameters. Based on these studies, it is difficult to predict how macroalgae will respond to OA in natural ecosystems. Moreover, the relevance of short-term studies to understand longer-time scale adaptive responses is questionable. Consequently, there is an increasing interest to assess adaptive response and potential of marine organisms to face climate change stressors over longer-time scales (Lohbeck et al., 2012; Sunday et al., 2014; Hutchins et al., 2015; Stillman and Paganini, 2015). The most direct approach to address adaptive responses consists of multi-generational evolution experiments performed on microorganisms with short generation times (Collins and Bell, 2004; Lohbeck et al., 2012; Benner et al., 2013). Macroalgae are not suitable for such approach due to their longer life cycles and their strong interactions with other ecosystem components, which are difficult to simulate under controlled conditions used in laboratory experiments, microcosm and mesocosms studies.

In this context, the shallow underwater volcanic vents with naturally acidified waters around the Castello Aragonese off the Ischia Island (Gulf of Naples; Hall-Spencer et al., 2008) offer a unique opportunity to investigate the effects of OA. Variation in the occurrence of these vents established three contrasting zones, characterized by pH values of 8.14 ± 0.01, 7.83 ± 0.06, and 6.72 ± 0.06, respectively (Porzio et al., 2011). At the lowest pH site on rocky substrate, from 0.70 to 1.0 m below mean sea level, the algal cover is dominated by the fucoid alga *Sargassum vulgare*, whose settlement dates back at least three decades (Porzio et al., 2017). Fucoid algae release synchronously gametes in calm sea conditions, they mate in close vicinity and their propagules have a low dispersal rate (Kendrick and Walker, 1991; Pearson and Serrão, 2006). Therefore, it can be assumed that the population settled at low pH at these volcanic vents is genetically relatively isolated. This provides an ideal set-up to study its long-term response to acidification in the natural habitat. Recently, through a *de novo* transcriptome analysis, we revealed that this *S. vulgare* population is adapted to live at lowered pH (Kumar et al., 2017).

In order to extend these measurements and to understand the physiological and biochemical mechanisms responsible for adaptive and stress responses of *S. vulgare* to OA, we analyzed photosynthesis, oxidative stress levels, antioxidant contents, antioxidant enzyme activities, and activities of oxidative metabolic enzymes in natural populations as well as in *in situ* reciprocal transplants from control to acidified site and *vice-versa*.

## Materials and Methods

### Study Site and Sample Collection

**FIGURE 1:**
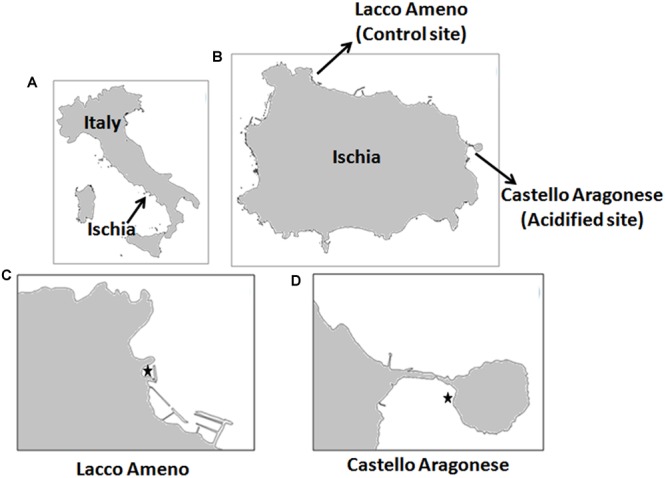
**Study sites. (A)** Map of Italy; **(B)** map of Ischia Island showing the location of Lacco Ameno and Castello Aragonese sites; **(C)** close up view of the control site Lacco Ameno; **(D)** close up view of the acidified site Castello Aragonese. In **(C,D)**, marked point showing locations of *Sargassum vulgare* (Image generated through qGIS v. 2.12.2).

*Sargassum vulgare* plants were collected along the coast of the Ischia Island at two locations: Castello Aragonese (acidified site, 40°43.87N, 013°57.78E) and Lacco Ameno (control site, 40°45.35N, 013°53.13E) (**Figure 1**). Castello Aragonese is the site where underwater CO_2_ vents lower the local pH. These venting activities date back to nearly 2000 years (Lombardi et al., 2011), releasing gases mainly constituting CO_2_ (90.1–95.3%) in absence of harmful sulfur gas or effects on seawater temperature (Hall-Spencer et al., 2008). Variation in the occurrence of these vents established three contrasting zones characterized by pH values of 8.14 ± 0.01, 7.83 ± 0.06, and 6.72 ± 0.06, respectively (Porzio et al., 2011). The venting activities are variable at the hour scale, but on average the pH values in the most acidified zone are constantly around 6.7. Only in this area *S. vulgare* is settled and characterizes the algal community with one of the highest cover. Lacco Ameno is the control site, located about 6 km far northwest from Castello Aragonese with an average pH value of around 8.2 close to normal seawater, but with similar hydrodynamic and physical conditions as the acidified site. At both sites, *S. vulgare* populations are growing at similar depth (<1 m), wave exposition (sheltered bays), PAR transmission properties of the water, temperature and salinity (Kumar et al., 2017). pH (NBS scale) and temperature were measured in triplicate at the collection time, as reported in Kumar et al. (2017).

*In situ* reciprocal transplants were performed in July. Five individuals of *S. vulgare* originating from the control site (C) in Lacco Ameno were tied in a net and moved to the acidified site (A) in Castello Aragonese (C-A), and *vice-versa* (A-C). In order to evaluate the stress effect due to the transplant itself, other thalli were also transplanted in their respective natural site (C-C and A-A) and used as controls.

Photosynthetic parameters were measured *in situ* using a Diving-PAM (Pulse Amplitude Modulated) fluorometer (Walz, Effeltrich, Germany) on natural populations from both sites and on the reciprocal transplants (2 weeks after transplantation). To avoid differences due to variable environmental conditions, all analyses were performed on the same day between 11 am and 1 pm.

The samples for biochemical analyses on natural populations were collected in March and July on the same day at approximately the same time (between 11 am and 1 pm) in order to avoid effects of environmental fluctuations other than pH and CO_2_. In both locations, a total of nine thalli of similar size (8–10 cm frond length) were handpicked in three different patches by snorkeling along a coastal stretch of 15 m to cover the natural variability of the two local populations. For the analyses on *in situ* reciprocal transplants, samples were collected in the same way at 2 weeks after transplantation.

The collected samples were maintained onboard in water of their respective sites and brought to the laboratory where the tissues were washed with filtered sea water, using a soft paint brush to remove visible epiphytes. The samples were either processed immediately or snap frozen in liquid nitrogen and stored at -80°C for further analysis.

### pH Drift Experiment

A preliminary experiment was conducted in the lab in order to determine the ability of the two populations of *S. vulgare* (settled in the control and the acidified site) to use ${\mathrm{HCO}}_{3}^{–}$ as a carbon source using the method developed by Hepburn et al. (2011). If the algae are able to increase the pH above 9, it means they are able to utilize ${\mathrm{HCO}}_{3}^{–}$, because at higher pH the concentration of CO_2_ is so low that it limits photosynthesis for obligate CO_2_ using macroalgae (Cornwall et al., 2012). To test this, eight independent algal thalli (1 g fresh weight each; four from control site and four from acidified site) were cleaned and placed into a 50 ml sealed transparent container containing sterile seawater (passed through 0.22 μm filters, followed by UV treatment). The incubation was done in seawater at two different pH values (pH 8.1 representing normal seawater and pH 7.0, the value recorded in seawater taken directly from volcanic CO_2_ vents at the time of harvest). Actinic light was supplied at a level of ca. 200 μmol photons m^-2^s^-1^ at 25°C. After 24 h, the algae were removed from the containers and pH was recorded. The containers were left open for 24 h before the pH was measured again.

### Photosynthetic Parameters and Pigment Analysis

The collection and processing of fluorescence data, obtained using a Diving-PAM fluorometer (Walz, Effeltrich, Germany), were performed fundamentally following the guidelines suggested by Ralph and Gademann (2005). Rapid light curves (RLCs) of irradiance vs. electron transport rate (ETR, past PSII) were obtained by exposing algal thalli spots to a range of irradiances between ∼ 13 and 400 μmol photons m^-2^ s^-1^, produced by the Diving-PAM lamp, and lasting 10 s after each 10 min dark adaptation period. Photosynthetic parameters, such as relative maximum electron transport rate (rETRmax), initial slope of the curve (α), and the saturating irradiance (Ek) were calculated by fitting empirical data to an exponential function of Webb et al. (1974). We also estimated the maximum photosynthetic efficiency of PSII (*F*_v_/*F*_m_) of the two populations. Chlorophyll a and c content was determined in tissues extracted in 80% acetone according to Mitchell and Kiefer (1984).

### Determination of Oxidative Stress Markers and Antioxidant Enzyme Activities

Intracellular ROS in the algal tissues were quantified by measuring the oxidation of 2′,7′-dichlorohydrofluorescein diacetate (DCFH-DA, Sigma) according to Collen and Davison (1997). Hydrogen peroxide (H_2_O_2_) content was measured in the frozen algal tissues by the FOX1 assay, based on the peroxide mediated oxidation of Fe^2+^, followed by reaction of Fe^3+^ with xylenol orange. Absorbance of the Fe^3+^-xylenol orange complex was measured at 560 nm (Jiang et al., 1990). Lipid peroxidation was assessed by monitoring the production of malondialdehyde (MDA), according to Hodges et al. (1999). Total antioxidant capacity was measured using ferric reducing/antioxidant power (FRAP) assay, according to Benzie and Strain (1998). Ascorbate (ASC) and glutathione (GSH) were quantified by extraction of frozen algal tissue in ice cold 6% metaphosphoric acid and analysed on a reversed phase HPLC column (100 mm × 4.6 mm Polaris C18-A, 3 μm particle size) at 40°C with an isocratic flow rate of 1 ml min^-1^ of elution buffer (2 mM KCl, pH 2.5 adjusted with *o*-phosphoric acid). Total ASC (ASCt) and GSH (GSHt) concentrations (reduced + oxidized) were determined after reduction with 0.04 M DTT for 10 min at room temperature according to Potters et al. (2004). The redox states (ASC redox state and GSH redox state) were calculated as the reduced form to the total concentration ratio. Total polyphenols and flavonoids were extracted in 80% ethanol. Phenolic content was measured by Folin Ciocalteu assay according to Zhang et al. (2006), with gallic acid as standard. Flavonoid content was measured by modified aluminum chloride colorimetric method according to Chang et al. (2002), with quercetin as standard. Tocopherols were extracted by homogenizing algal tissue in hexane, and quantified by HPLC analysis according to Siebert (1999). Data were analyzed with Shimadzu Class VP 6.14 software provided by the HPLC system (Shimadzu, Tokyo, Japan).

For antioxidant enzymatic assays, protein extracts were prepared according to Murshed et al. (2008) and quantified according to Lowry et al. (1951). All enzyme activities were determined in 200 μL volume kinetic reactions at 25°C, using a micro-plate reader. APX, dehydroascorbate reductase (DHAR), monodehydroascorbate reductase (MDHAR), glutathione reductase (GR) activities were determined according to Murshed et al. (2008). Peroxidase (POX) activity was determined according to Kumar and Khan (1982). SOD activity was determined according to Dhindsa et al. (1981). CAT activity was determined according to Aebi (1984). GPX activity was determined according to Drotar et al. (1985). GST activity was determined according to Habig et al. (1974). Peroxiredoxin (PRX) activity was determined according to Horling et al. (2003). Glutaredoxin (GRX) activity was determined according to Lundberg et al. (2001). Thioredoxin (TRX) activity was determined according to Wolosiuk et al. (1979). Ferredoxin-NADP(H) Reductase (FNR) activity was determined according to Rodriguez et al. (2007). All the oxidative stress markers and antioxidant enzymatic activities were determined on three or five (ROS measurements) independent specimens.

### Determination of Enzymatic Activities Related to Energy Metabolism

The activities of NADH dehydrogenase (NADH-DH) and cytochrome c oxidase (COX) were measured in the algal tissues (*n* = 3) by spectrophotometric methods. NADH-DH activity was measured according to Galante and Hatefi (1978) by using a modified reaction mixture (1 ml) containing 50 mM phosphate buffer (pH 7.4), 0.1% Triton X-100 (v/v), 1.6 mM potassium ferricyanide, 0.17 mM NADH, and 30 μg mitochondrial protein in phosphate buffer. This reaction mixture had slightly lowered pH value and contained lower and higher concentrations of NADH and potassium ferricyanide, respectively, as compared to the original protocol. Samples treated with L-3,4-dihydroxyphenylalanine (L-DOPA) to suppress NADH-DH activity were used as negative controls. The absorbance was measured at 410 nm and NADH-DH activity was calculated using an extinction coefficient of 1 mM^-1^ cm^-1^. COX activity was determined according to Goyal and Srivastava (1995).

### Determination of Nitric Oxide and Protein Nitrosothiols

Nitric oxide levels in the algal tissues (*n* = 5) were measured with the Griess reagent according to Green et al. (1982). *S*-nitrosothiol content was determined according to Park and Kostka (1997).

### Statistical Analysis

Student’s *t*-tests were performed in order to assess differences between two study sites. Condition of homogeneity of variance was examined by Levien’s test. Independent sample *t*-test was performed on the data to determine the significant difference between the mean values. The analysis of differences between the control and the acidified site was independently performed for both seasons. For transplant experiments, significance was analyzed in samples transplanted from the control to the acidified site and *vice-versa*, compared to the respective controls transplanted to the same site. All statistical analyses were performed using SPSS v21 (SPSS Inc, Chicago, IL, USA).

## Results

### ${\mathrm{HCO}}_{3}^{–}$ as a Carbon Source

Samples collected from the acidified site and incubated for 24 h in sea water at pH 8.08 and 7.0 in closed containers raised the pH to 9.14 ± 0.01 SE and 9.05 ± 0.05 SE, respectively. Samples from the control site, treated following the same protocol, showed an increase of the pH values to 9.16 ± 0.03 SE and 9.1 ± 0.04 SE, respectively. The ability to raise pH above 9.0 demonstrates the capacity of *S. vulgare* to use ${\mathrm{HCO}}_{3}^{–}$ as carbon source in both conditions. After 24 h of removing algae, we observed that the pH dropped to the values of ca. 8.1 in all containers, indicating that chemical conditions of the seawater had been re-equilibrated with air. These measurements also assured that pH change was not affected by algal exudates.

### Photosynthetic Performance and Pigments

We did not observe any significant differences in the photosynthetic performance between algae naturally growing at the acidified and the control site, respectively. However, in the transplants there was a significant increase in rETRmax and Ek and a decrease in α in plants transplanted from the control to the acidified site in comparison to those transplanted in the same site. We also noticed a decrease in *F*_v_/*F*_m_ in samples transplanted from the acidified to the control site (**Table 1**). The concentration of chlorophyll *c* was higher in algae from the acidified site and in samples transplanted from the acidified to the control site (**Table 1**).

**Table 1:** Photosynthetic parameters and pigment contents in *S. vulgare* collected at the acidified **(A)** and the control **(C)** site (natural populations) and after reciprocal transplants: C to C, control to control; C to A, control to acidified; A to A, acidified to acidified; A to C, acidified to control.

	Natural populations		Transplants			
	C	A	C to C	C to A	A to A	A to C
rETRmax (μmol electrons m^-2^ s^-1^)	29.65 @ 0.45	34.61 @ 3.56	27.96 @ 3.39	39.16 @ 3.31ˆ*	34.27 @ 3.17	32.76 @ 1.42
α (μmol electrons m^-2^ s^-1^/μmol photons m^-2^ sec^-1^)	0.33 @ 0.02	0.33 @ 0.02	0.34 @ 0.02	0.26 @ 0.01ˆ*	0.26 @ 0.00	0.25 @ 0.01
Ek (μmol photons m^-2^ s^-1^)	91.81 @ 7.94	96.04 @ 5.23	84.96 @ 14.96	146.89 @ 8.47ˆ*	133.02 @ 13.56	138.85 @ 14.20
*F*_v_/*F*_m_	0.72 @ 0.014	0.74 @ 0.002	0.71 @ 0.013	0.71 @ 0.002	0.71 @ 0.006	0.60 @ 0.040ˆ*
Chlorophyll a (mg/g FW)	32.38 @ 2.62	36.29 @ 1.25	24.72 @ 2.00	23.32 @ 1.89	27.71 @ 0.95	29.65 @ 1.02
Chlorophyll c (c_1_+c_2_) (mg/g FW)	13.23 @ 0.42	19.04 @ 0.22ˆ***	10.10 @ 0.32	9.53 @ 0.30	14.54 @ 0.17	15.56 @ 0.18ˆ*

*Values are mean ± SE, *n* = 5, ^∗^*p* < 0.05, ^∗∗∗^*p* < 0.001.*

### Redox State

**FIGURE 2:**
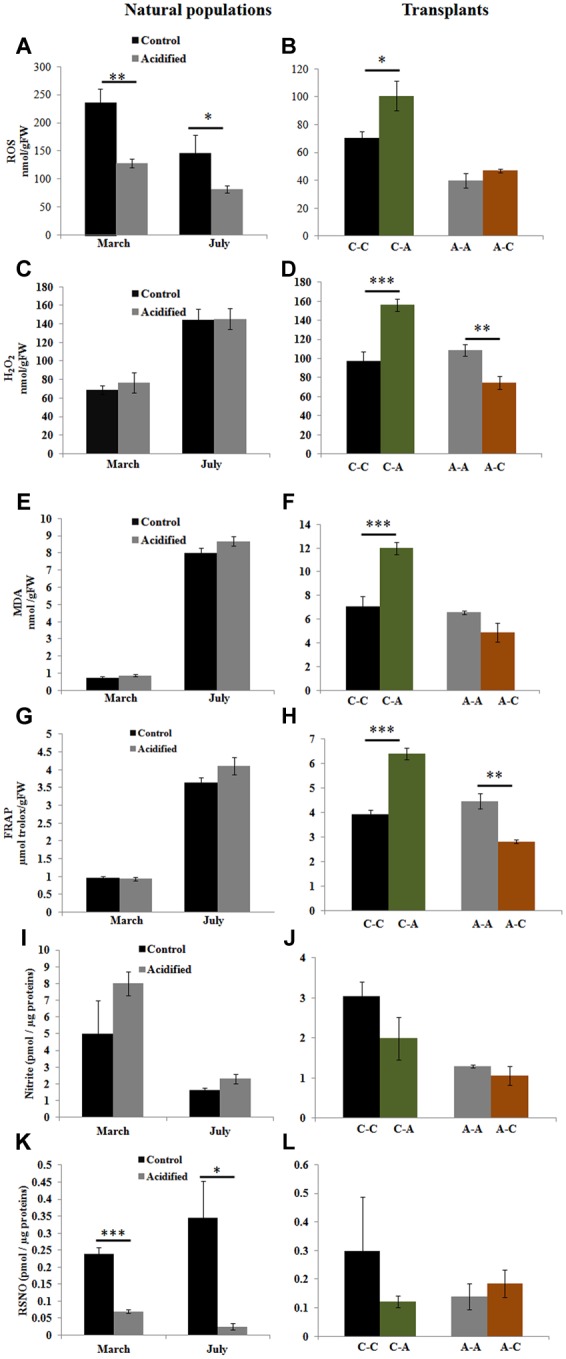
**Reactive oxygen species (ROS), hydrogen peroxide (H_2_O_2_), lipid peroxidation [malondialdehyde (MDA)], total antioxidant capacity [ferric reducing/antioxidant power (FRAP) assay], nitric oxide (nitrite), and protein nitrosothiol (RSNO) levels in *S. vulgare* samples collected at the control and the acidified site in March and July (natural populations) (A,C,E,G,I,K)** and in transplants **(B,D,F,H,J,L)**. C-C = control to control, C-A = control to acidified, A-A = acidified to acidified, A-C = acidified to control. Values are mean ± SE, *n* = 3 (*n* = 5 for ROS, nitric oxide, and RSNO), ^∗^ *p* < 0.05, ^∗∗^ *p* < 0.01, ^∗∗∗^ *p* < 0.001.

In order to understand if OA induces oxidative and nitrosative stress in *S. vulgare*, we examined the cellular redox state by measuring ROS, H_2_O_2_, lipid peroxidation, total antioxidant capacity, nitric oxide, and protein nitrosothiol levels. Total ROS was lower in the algae grown at the acidified site compared to those from the control site (**Figure 2A**), but no differences in H_2_O_2_, MDA, and total antioxidant capacity were observed between the two sites (**Figures 2C,E,G**). In samples transplanted from control to acidified site, there was a significant increase in total ROS, H_2_O_2_, MDA, and total antioxidant capacity (**Figures 2B,D,F,H**). Inversely, H_2_O_2_ and total antioxidant capacity decreased in algae transplanted from acidified to control site (**Figures 2D,H**). No significant variation was observed in nitric oxide levels (**Figures 2I,J**), whereas lower levels of *S*-nitrosylated proteins were detected in algae living at the acidified site compared to those of the control site (**Figure 2K**). No changes in nitrosylation were found in transplants (**Figure 2L**).

As the total antioxidant capacity reflects overall changes in small molecular antioxidants, we separately quantified the levels of the major antioxidant molecules, including ASC, GSH, tocopherols, polyphenols, and flavonoids (**Table 2**). ASCt and ASC redox states were generally lower in the acidified site; however, the difference was significant only in samples collected in March. ASCt was increased in samples transplanted from the acidified to the control site. GSHt and GSH redox states were affected only in natural samples collected in March only. The level of α-tocopherol was higher in the algae at the acidified site in both seasons. α-tocopherol levels consistently increased in transplants from the control to the acidified site and decreased in samples transplanted from the acidified site to the control conditions. β- and γ-tocopherols were increased only in samples transplanted from the acidified to the control site. Polyphenol and flavonoid levels were lower in the algae living for a long time at the CO_2_ vents site. Moreover, polyphenol levels decreased when algae were transferred from the acidified to the control site.

**Table 2:** Concentrations of ASCt, GSHt, ascorbate (ASC) redox state, and glutathione (GSH), tocopherols (alfa toc, beta toc, gamma toc), polyphenols and flavonoids in *Sargassum vulgare* collected at the acidified (A) and the control (C) site in March and July (natural populations) and after reciprocal transplants: C to C = control to control, C to A = control to acidified, A to A = acidified to acidified, A to C = acidified to control.

	Natural populations				Transplants			
	March		July					
	C	A	C	A	C to C	C to A	A to A	A to C
ASCt (μmol/gFW)	0.22 @ 0.01	0.14 @ 0.01ˆ*	0.48 @ 0.12	0.33 @ 0.02	0.64 @ 0.21	0.40 @ 0.08	0.37 @ 0.01	0.713 @ 0.086ˆ*
ASC redox state (%)	93.83 @ 1.77	72.78 @ 7.54ˆ*	81.03 @ 6.94	62.74 @ 8.42	86.13 @ 6.64	73.46 @ 3.08	55.05 @ 11.24	55.05 @ 11.24
GSHt (μmol/gFW)	0.03 @ 0.002	0.04 @ 0.00ˆ*	0.12 @ 0.03	0.08 @ 0.01	0.15 @ 0.05	0.100 @ 0.01	0.08 @ 0.01	0.110 @ 0.023
GSH redox state (%)	72.90 @ 2.70	49.50 @ 0.17ˆ*	55.43 @ 9.58	61.93 @ 3.98	57.14 @ 7.96	61.93 @ 3.97	47.90 @ 7.30	62.03 @ 0.74
Alfa toc (ug/gFW)	8.82 @ 0.86	13.44 @ 0.80ˆ**	30.10 @ 3.55	41.32 @ 2.49ˆ*	19.26 @ 2.27	28.86 @ 1.95ˆ*	26.34 @ 1.67	14.98 @ 1.09ˆ*
Beta toc (ug/gFW)	0.88 @ 0.07	1.11 @ 0.09	2.37 @ 0.18	2.99 @ 0.26	1.65 @ 0.11	2.32 @ 0.40	2.14 @ 0.35	4.20 @ 0.24ˆ*
Gamma toc (ug/gFW)	0.74 @ 0.18	0.56 @ 0.08	1.54 @ 0.26	0.21 @ 0.04	1.10 @ 0.19	1.87 @ 0.22	1.69 @ 0.25	4.62 @ 0.20ˆ*
Polyphenol (μmol GA/gFW)	2.37 @ 0.28	1.41 @ 0.22ˆ*	3.53 @ 0.41	2.24 @ 0.12ˆ*	6.25 @ 0.16	6.01 @ 0.13	4.13 @ 0.10	3.09 @ 0.06ˆ*
Flavonoids (mmol quercetin/gFW)	2.32 @ 0.45	1.45 @ 0.13	5.07 @ 0.90	2.55 @ 0.13ˆ*	2.50 @ 0.08	3.49 @ 0.28	2.03 @ 0.29	1.99 @ 0.20

*Values are mean ± SE, *n* = 3, ^∗^*p* < 0.05, ^∗∗^*p* < 0.01.*

### Antioxidant Enzyme Activities

**FIGURE 3:**
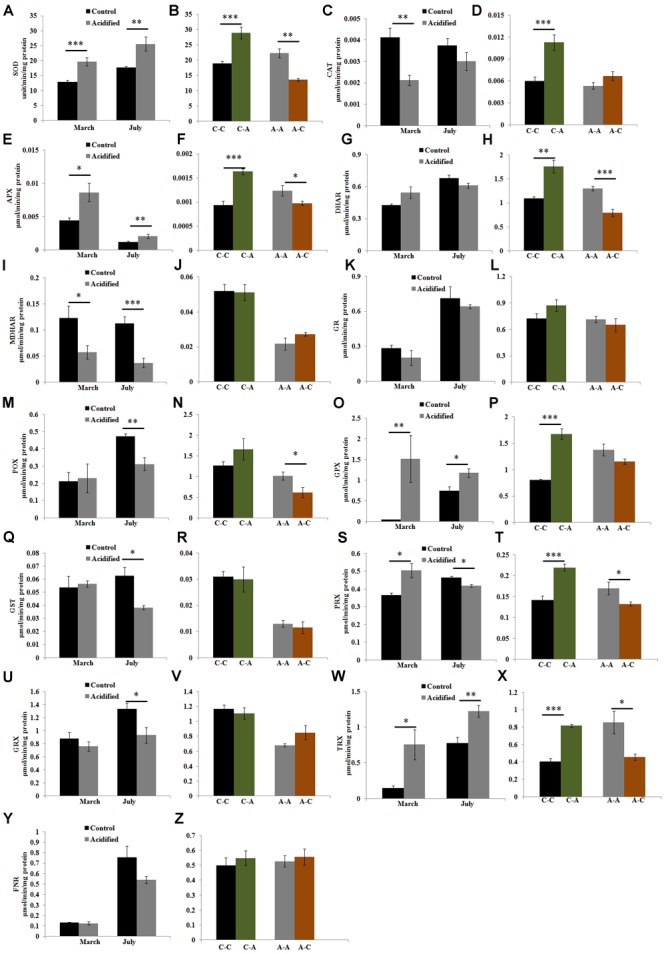
**Activities of antioxidant enzymes [superoxide dismutase (SOD), catalase (CAT), ascorbate peroxidase (APX), dehydroascorbate reductase (DHAR), monodehydroascorbate reductase (MDHAR), glutathione reductase (GR), Peroxidase (POX), glutathione peroxidase (GPX), glutathione *S*-transferase (GST), Peroxiredoxin (PRX), Glutaredoxin (GRX), Thioredoxin (TRX), and Ferredoxin-NADP(H) Reductase (FNR)] in *S. vulgare* samples collected at the control and the acidified site in March and July (natural populations) (A,C,E,G,I,K,M,O,Q,S,U,W,Y)** and after reciprocal transplants **(B,D,F,H,J,L,N,P,R,T,V,X,Z)**. C-C = control to control, C-A = control to acidified, A-A = acidified to acidified, A-C = acidified to control. Values are mean ± SE, *n* = 3, ^∗^ *p* < 0.05, ^∗∗^ *p* < 0.01, ^∗∗∗^ *p* < 0.001.

To understand the mechanisms responsible for the maintenance of the cellular redox state in the algae living under contrasting conditions, we investigated the enzymatic components of the antioxidant machinery. SOD activity was higher in samples at the acidified site (**Figure 3A**) and in samples transplanted from the control to the acidified site, while the activity decreased in transplants from the acidified site to the control one (**Figure 3B**). The activities of H_2_O_2_ scavenging enzymes showed variable responses (**Figures 3C–Z**). In algae living at the acidified site, the activities of APX, GPX, and TRX were higher in both seasons compared to control samples, while in the case of PRX higher values were observed only in March samples. Inversely, levels of CAT, MDHAR, POX, GST, PRX, and GRX activities in samples collected at the acidified site were lower than the control samples. This trend was observed in both seasons for MDHAR, only in March for CAT, and only in July for the other enzymatic activities (POX, GST, PRX, and GRX). Upon short-term transplants of algae from the control to the acidified site, an increase in the activities of CAT, APX, DHAR, GPX, PRX, and TRX was observed. Inversely, APX, DHAR, POX, PRX, and TRX activities decreased when samples were moved from the acidified to the control site.

### Oxidative Metabolism

**FIGURE 4:**
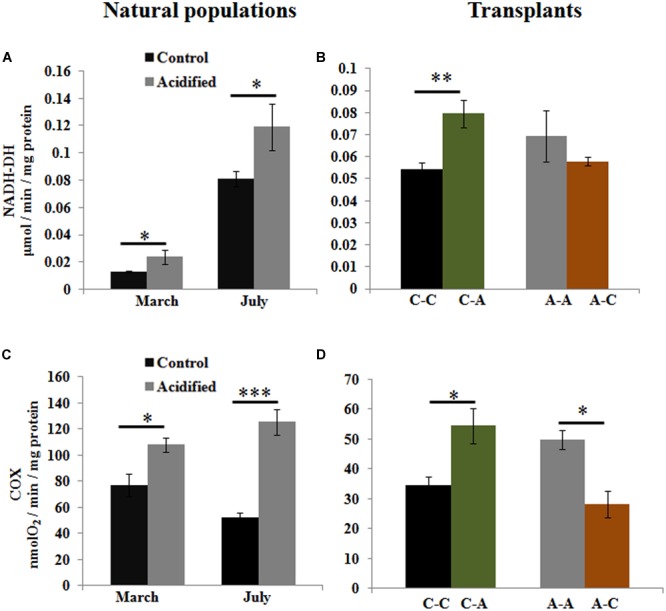
**Activities of oxidative metabolic enzymes, NADH-DH, and cytochrome c oxidase (COX), in *S. vulgare* samples collected at the control and the acidified site in March and July (natural populations) (A,C)** and after reciprocal transplants **(B,D)**. C-C = control to control, C-A = control to acidified, A-A = acidified to acidified, A-C = acidified to control. Values are mean ± SE, *n* = 3, ^∗^ *p* < 0.05, ^∗∗^ *p* < 0.01, ^∗∗∗^ *p* < 0.001.

To examine whether OA affects the energy metabolism in this species, we measured the activities of oxidative metabolic enzymes. Higher activities of NADH-DH and COX were detected in algae living at the acidified site (**Figures 4A,C**). Transplantation from the control to the acidified site also caused increase of these enzymatic activities (**Figures 4B,D**), whereas the inverse transplantation caused a decrease in COX activity (**Figure 4D**).

## Discussion

The aim of this study was to understand the molecular mechanisms responsible for the survival of the brown alga *S. vulgare* under increased CO_2_ levels that lead to acidification. Both effects are linked, also when induced by global climate change, and are likely to affect the growth and survival of key algal species. Therefore, it is important to understand how these environmental changes affect dominant macroalgae under natural conditions in long- and short-term. Therefore, following a genome-wide transcriptome analysis (Kumar et al., 2017), we addressed physiological and biochemical parameters to understand the long- and short-term adaptation of *S. vulgare* populations naturally growing in acidified conditions.

### Photo-Physiological Responses

The lack of significant changes in the photosynthetic parameters of the *S. vulgare* population living at the vents site of the Castello Aragonese (Ischia Island) could be related to the presence of carbon concentration mechanisms which rely on ${\mathrm{HCO}}_{3}^{–}$ utilization (Koch et al., 2013). The results of the pH drift experiments indicated that the main exogenous inorganic carbon source for *S. vulgare* was indeed ${\mathrm{HCO}}_{3}^{–}$. Moreover, even though there would be more diffusive entry of CO_2_ into the cells under acidified conditions, the capacity for ${\mathrm{HCO}}_{3}^{–}$ utilization was not affected. Similar effects on photosynthesis were obtained in other long-term studies conducted *in situ* on the brown alga *Lobophora variegata* (Betancor et al., 2014) and the seagrass *Posidonia oceanica (L.) Delile* (Hall-Spencer et al., 2008). In contrast, an increase in rETRmax was detected in *Cystoseira corniculata* (Baggini, 2015) and in the calcifying species *Padina pavonica and P. australis* at volcanic seeps in Papua New Guinea (Johnson et al., 2012). Our short-term data from *S. vulgare* transplant experiments (from the control to the acidified site) revealed an increase in rETRmax, in line with the positive photophysiological response observed in *Cystoseira compressa* after short-term transplant at CO_2_ vents off the island of Vulcano (Celis-Plá et al., 2015). The finding that short-term exposure to acidification induced photosynthetic responses suggests a physiological acclimatization in *S. vulgare*. On the other hand, the absence of changes in photosynthesis under conditions of chronic acidification indicates that the local population is adapted. Initially, increased levels of CO_2_ might be beneficial for *S. vulgare*, allowing them to grow faster. Under acute increased CO_2_ levels, photosynthesis may no longer be CO_2_-limited, and CCMs unnecessary, allowing the algae to saved energy that can be allocated to growth, explaining their dominance around volcanic CO_2_ vents in the Mediterranean Sea (Porzio, 2010; Baggini et al., 2014). Furthermore, our reciprocal transplants from the acidified to the control pH showed signs of physiological stress (decreased *F*_v_/*F*_m_), suggesting again that the algal population at the vents is adapted to grow under the acidified conditions.

### Energy Metabolism

It should be kept in mind that, apart from constituting a benefit for autotrophs, the increase in aqueous CO_2_ causes seawater acidification which has the potential to affect metabolism and cellular homeostasis, thereby impairing cellular function or increasing energy demand, as it has been found in cyanobacteria and phytoplankton (Taylor et al., 2012; Brutemark et al., 2015). Indeed, we found higher activities of oxidative metabolizing enzymes in response to short-term transplants from the control to the acidified site; these higher values were maintained in natural populations under long-term conditions of acidification. This finding is in line with our recent RNA Seq data showing up-regulation of the transcripts encoding proteins involved in energy metabolism, such as NADH dehydrogenase subunits I, II, IV, and cytochrome oxidase subunits I, II, III, in *S. vulgare* growing for long-term at Ischia CO_2_ vents (Kumar et al., 2017). A general decrease in activities of oxidative enzymes upon transplantation of *S. vulgare* from acidified to control conditions further supported our finding that acidification increases energy demand.

### Cellular Redox Status

Notwithstanding elevated CO_2_ can induce oxidative stress in marine animal organisms (Tomanek et al., 2011; Hu et al., 2015), no data are available for macroalgae. To our knowledge, this is the first study monitoring redox state and antioxidant activities in macroalgae in response to *in situ* acidification. We observed that thalli of *S. vulgare* growing for a long time at pH conditions lower than current ones have developed mechanisms to maintain cellular redox homeostasis. In contrast, the imbalance of the redox state in thalli transplanted from the control to the acidified site, suggests that acidification induces stress in short-time scale. In autotrophs, respiration, and photosynthesis are sources of radical formation (Gill and Tuteja, 2010) and both processes were observed to be increased in *S. vulgare* thalli transplanted to the acidified site. Even though there was an increase in antioxidant capacity in plants transplanted from control to acidified conditions, it was most likely insufficient to balance the increased formation of oxidant species, leading to oxidative damage as indicated by increased levels of MDA (**Figure 2F**). However, in natural populations under long-term conditions ROS values were lower in the acidified site than those in the control one, whilst H_2_O_2_ and lipid peroxidation were comparable. These results could be explained with a higher efficiency of energy transfer reactions, suggesting the capability of the acidified population to overcome the negative effects of lowered pH and to sustain itself in the future acidified ocean, if acclimatized for longer periods. This is also supported by the finding that the levels of nitric oxide, which has been reported to be involved in different physiological responses in marine photosynthetic organisms (Kumar et al., 2015), do not change in natural populations from the acidified and the control sites as well as in transplants. On the other hand, OA induced reduction of protein *S*-nitrosylation, thus suggesting the modulation of nitric oxide signaling in adaptation of *S. vulgare*.

**FIGURE 5:**
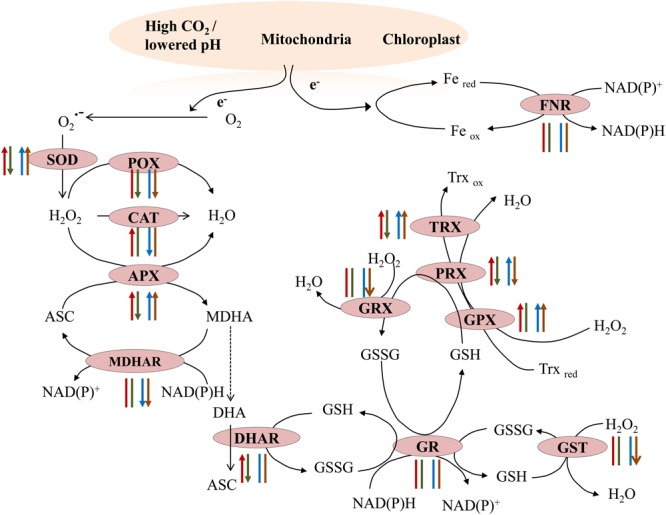
**Summary of antioxidant enzymes activity in natural populations and in transplants.** Transplants: control to acidified (red arrow), acidified to control (green arrow); natural populations: March (blue arrow), July (brown arrow). Arrow head up: increased activity; arrow head down: decreased activity; arrow without head: no changes in the activity.

The total antioxidant capacity, which determines the additive antioxidant properties of plants, was comparable in specimens from the two sites, but it increased in short-term transplants from control to acidified conditions and decreased in the opposite transplants. The total antioxidant activities have been reported to increase in short-term acidified conditions in the macroalga *C. compressa* (Celis-Plá et al., 2015) and in the microalga *Nannochloropsis salina* (Yangüez et al., 2015), while, in long-term conditions, a significant decrease has been reported for the brown alga *L. variegata* (Betancor et al., 2014). Data obtained in the present study also indicate that thalli of *S. vulgare* growing under acidified conditions for short-term will have a general increase in antioxidative enzyme activities. In natural populations under long-term conditions, some antioxidant activities retain high values to increase algal surviving capabilities, while others appeared unchanged or lower compared to controls, thus suggesting an adaptation process (**Figure 5**). SOD represents the first line of antioxidant defense in marine algae (Carvalho et al., 2004), and the induction of this enzyme indicates its key role in inhibiting superoxide radical overproduction upon an increase of oxidative metabolism under acidified conditions. The increase of SOD activity in transplant experiments from the control to the acidified site and its decrease in the reverse experiments are in line with a higher energy metabolism found after lower pH exposure. The accumulated H_2_O_2_ in *S. vulgare* in short-term acidification may be removed by increased CAT activity. The ascorbate-glutathione (ASC/GSH) cycle, a major mechanism of H_2_O_2_ control in autotrophs (Foyer and Noctor, 2011), which in higher plants alleviates stress impact by CO_2_ enrichment (AbdElgawad et al., 2015), showed also some changes in *S. vulgare* under acidified conditions. The increase in APX activity in both natural and transplanted samples was paralleled by slight decrease in ASC levels and the ASC redox state. In short-term transplants, acidification induced DHAR activity in *S. vulgare*, which is involved in generation of ASC. In natural populations under long-term conditions, MDHAR activity was decreased, while SOD and APX activities increased. These results are in line with our recent RNA Seq data showing increased transcription of SOD and APX, and down-regulation of MDHAR expression in populations living at the acidified site (Kumar et al., 2017). The finding under acidified conditions of the increased activities of the enzymes involved in H_2_O_2_ detoxification, PRX, TRX, and GPX (Foyer and Shigeoka, 2011), indicate an active participation of the thioredoxin dependent pathway of H_2_O_2_ removal in *S. vulgare.*

## Conclusion

The different response of *S. vulgare* to OA to long- and short-term exposures confirms that the population facing chronic acidification is adapted to live under acidified conditions. In reciprocal transplant experiments, *S. vulgare* acclimatized to acidification showed physiological stress (decreased *F*_v_/*F*_m_) when transferred to the control site, further supporting the idea that populations living for some decades at the vents site are adapted to grow under acidified conditions. The occurrence of oxidative stress in short-term conditions suggests that macroalgae need longer time to overcome the effects of acidification. However, utilizing molecular and enzymatic antioxidants, *S. vulgare* is capable to mitigate stress effects and adapt to acidified conditions.

**FIGURE 6:**
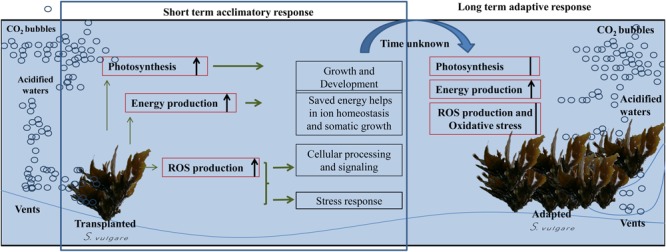
**Summary of possible series of events happened to *S. vulgare* at the acidified site.** Red boxes: examined processes; black boxes: hypothesized processes.

Based on these results, a series of events have likely happened in *S. vulgare* at the acidified site (**Figure 6**). The increase in the photosynthetic performance, and a higher energy production would be useful in maintaining ion-homeostasis and for enhancing growth. ROS, nitric oxide, and other redox molecules would be contributing toward maintaining cellular signaling and genetic regulation under acidified conditions. The short-term acclimation responses seem to allow *S. vulgare* to adapt to the acidified conditions, resulting in a population with a more active energy metabolism, without signs of oxidative stress and changes in photosynthetic efficiency. Overall, the results obtained in this study suggest that *S. vulgare* could be expected to be among the species benefitting from future acidified ocean.

## Author Contributions

AK, AP, MCB, IC, and MD designed the study, AK and MCB performed sample collection and *in situ* transplant experiments, AK and ML performed *in situ* photosynthetic measurements, AK and HAE performed biochemical and physiological experiments and collected data, AK, HAE, IC, MCB, and AP analyzed output data and results, GB and HA provided Materials and Methods for biochemical tests, AK wrote the first draft of the manuscript, and all the authors contributed substantially to the interpretation and final version of the paper.

## Conflict of Interest Statement

The authors declare that the research was conducted in the absence of any commercial or financial relationships that could be construed as a potential conflict of interest.
